# KRAS-mutation status dependent effect of zoledronic acid in human non-small cell cancer preclinical models

**DOI:** 10.18632/oncotarget.12806

**Published:** 2016-10-21

**Authors:** István Kenessey, Krisztina Kói, Orsolya Horváth, Mihály Cserepes, Dávid Molnár, Vera Izsák, Judit Dobos, Balázs Hegedűs, József Tóvári, József Tímár

**Affiliations:** ^1^ 2^nd^ Department of Pathology, Semmelweis University, Budapest, Hungary; ^2^ National Cancer Registry, National Institute of Oncology, Budapest, Hungary; ^3^ Department of Experimental Pharmacology, National Institute of Oncology, Budapest, Hungary; ^4^ Institute of Enzymology, Research Centre for Natural Sciences, Hungarian Academy of Sciences, Budapest, Hungary; ^5^ Vichem Chemie Ltd., Budapest, Hungary; ^6^ Hungarian Academy of Sciences-Semmelweis University Molecular Oncology Research Group, Budapest, Hungary

**Keywords:** zoledronic acid, ras inhibitor, human non-small cell lung cancer, xenograft model

## Abstract

**Background:**

In non-small cell lung cancer (NSCLC) KRAS-mutant status is a negative prognostic and predictive factor. Nitrogen-containing bisphosphonates inhibit prenylation of small G-proteins (e.g. Ras, Rac, Rho) and thus may affect proliferation and migration. In our preclinical work, we investigated the effect of an aminobisphosphonate compound (zoledronic acid) on mutant and wild type KRAS-expressing human NSCLC cell lines.

**Results:**

We confirmed that zoledronic acid was unable to inhibit the prenylation of mutant K-Ras unlike in the case of wild type K-Ras. In case of *in vitro* proliferation, the KRAS-mutant human NSCLC cell lines showed resistance to zoledronic acid wild-type KRAS-cells proved to be sensitive. Combinatory application of zoledronic acid enhanced the cytostatic effect of cisplatin. Zoledronic acid did not induce significant apoptosis. In xenograft model, zoledronic acid significantly reduced the weight of wild type KRAS-EGFR-expressing xenograft tumor by decreasing the proliferative capacity. Futhermore, zoledronic acid induced VEGF expression and improved *in vivo* tumor vascularization.

**Materials and methods:**

Membrane association of K-Ras was examined by Western-blot. *In vitro* cell viability, apoptotic cell death and migration were measured in NSCLC lines with different molecular background. The *in vivo* effect of zoledronic acid was investigated in a SCID mouse subcutaneous xenograft model.

**Conclusions:**

The *in vitro* and *in vivo* inhibitory effect of zoledronic acid was based on the blockade of cell cycle in wild type KRAS-expressing human NSCLC cells. The zoledronic acid induced vascularization supported *in vivo* cytostatic effect. Our preclinical investigation suggests that patients with wild type KRAS-expressing NSCLC could potentially benefit from aminobisphosphonate therapy.

## INTRODUCTION

Lung cancer is the lead neoplastic cause of death, furthermore, both incidence and mortality show constant elevation [1]. As one of the most aggressive neoplastic diseases, overall survival including stage III and stage IV patient groups is less than 5% within five years [2]. Treatment opportunities for non-small cell lung cancer (NSCLC) were very limited, traditionally the first option was surgical resection, where available, complementing cisplatin-based cytostatic therapy [3]. The situation has completely changed with the appearance of target based therapy: small molecule EGFR-TKIs (e.g. gefitinib, erlotinib) have effects on cancer cells that carry the activating mutation of EGFR [4]. Nevertheless, disorders in the EGFR-signal can impact the efficacy of chemotherapy. For instance, mutant KRAS is a negative prognostic and predictive factor of both classic and target based therapy, links to poor survival and increased progression. Moreover, adenocarcinomas expressing mutant KRAS show resistance against small tyrosine kinase inhibitors, gefitinib and erlotinib [4]. According to the recent guidelines, before administration of EGFR-TKI the mutational status of KRAS as well as EGFR is required to be examined [5].

The mutation of RAS-oncogene can be found in a variety of tumor types, its frequency is 30% in lung cancers [6]. Ras proteins are small GTP-binding proteins that affect cell proliferation, migration and survival [7]. The activating mutations of KRAS affect three of the four EGFR-pathways, the PI3K-, BRAF- and PLC-signals [8]. The active wild type Ras is localized in the plasma membrane, which requires prenylation by farnesyltransferases or geranylgeranyltransferases [9]. This posttranslational modification might be blocked by inhibition farnesyl-diphosphate synthase, the key enzyme of cholesterol synthesis [10]. Prenylation-inhibitory nitrogen-containing bisphosphonates (NBPs) and statins are promising candidates for clinical therapy of patients with cancer, albeit originally other indications were accepted [11]. Nitrogen-containing bisphosphonates (e.g. zoledronic acid) preventing bone-metastasis inhibit a key enzyme, farnesyl diphosphonate (FPP) synthase in the biosynthetic mevalonate pathway, which interferes with numerous essential functions of osteoclasts [12]. Several steps of this pathway are required for the post-translational modification of small G-proteins. These macromolecules (e.g. Ras, Rac, Rho) play crucial role in the regulation of cell proliferation, survival, and migration [13].

Numerous previous studies suggested the antitumor activities of NBPs not only in bone metastases but directly on the primary tumor [14]. Preclinical works proved that NBPs inhibited proliferation and induced apoptosis in human myeloma, breast cancer, pancreatic cancer, prostate cancer, small and non-small cell lung cancer, and osteosarcoma cell lines in dose- and time-dependent manner [15–21]. Furthermore, in combination zoledronic acid with traditional cytostatic agent, paclitaxel showed synergistic effect on breast cancer cells [22]. Many animal models supported direct anticancer effect of NBPs: combinatorial treatment enhanced the effect of cytostatic therapy on the growth of human primary breast cancer, pancreatic cancer and non-small cell cancer [23–25]. Moreover, isolated clinical cases were described the effect of zoledronic acid in monotherapy, for instance, in a patient with pulmonary adenocarcinoma, the treatment caused regression of the primary lesion as well as hepatic metastasis [26].

Previous *in silico* and *in vitro* studies confirmed the antitumor effect of NBPs on EGFR-driven cancers [27, 28], albeit beside the EGFR itself the inhibition of RAS is seemed to be still another important target. The exact mechanism of latter still remains unclear. In the present study we provide several lines of evidence that the antiproliferative effect of zoledronic acid depends on the KRAS-status of human NSCLC-lines, mutant KRAS showed resistance, while wild type KRAS could be inhibited *in vitro* as well as *in vivo* affecting proliferation and growth of the tumor. On the contrary, migratory capacity proved independent of KRAS-status of human NSCLC lines.

## RESULTS

### Zoledronic acid had different effects on mutant and wild type KRAS

**Figure 1:**
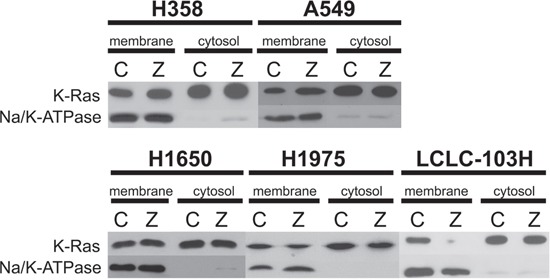
The effect of zoledronic acid on the prenylation of human NSCLC cell lines with mutant and wild type KRAS Cell suspensions were separated into membrane and cytosol fractions by ultracentrifuge. Western blot confirmed that compared to control (C), treatment with 25 μM zoledronic acid (Z) could not modify membrane-association of mutant K-Ras in H358 and A549, while in LCLC-103H prenylation of wild type K-Ras was inhibited. H1975 cells showed approx. 50 percentage decrease, while zoledronic acid did not inhibit prenylation of wild type K-Ras in H1650 cells. Na/K-ATPase was applied as loading control.

Prenylated and unprenylated form of K-Ras was detected in previously treated and control NSCLC cancer cell lines with different molecular background. LCLC-103H cells express wild type KRAS and wild type EGFR, A549 and H358 express mutant KRAS, H1650 cells express activated mutation in EGFR, H1975 cells express resistant mutation in EGFR (Table 1). Cell suspensions were separated into cytosol and membrane fractions by ultracentrifuge, latter represented and immunoblotting revealed that in A549 and H358 cells 25 μM zoledronic acid could not affect membrane-association of exon2-mutant K-Ras protein. In contrast, in double wild type LCLC-103H cells zoledronic acid treatment significantly reduced membrane association of K-Ras, as well as H1975 with resistance mutation in EGFR, nevertheless adjusting to Na/K-ATPase latter showed only 50% decrease. Surprisingly, EGFR-mutant cell line H1650 did not show effect on K-Ras (Figure 1).

**Table 1:** Oncogenic mutations in the applied human NSCLC-lines

NSCLC-lines	KRAS	EGFR
**LCLC-103H**	wt	wt
**A549**	G12S	wt
**H358**	G12C	wt
**H1650**	wt	del E746-A750
**H1975**	wt	L858R/*T790M*

H1975 is inherently resistant to EGFR inhibitors as it carries the resistance mutation T790M (in italics) besides the activating mutation L858R.

### Effect of zoledronic acid on the proliferation of human NSCLC cell lines

**Figure 2:**
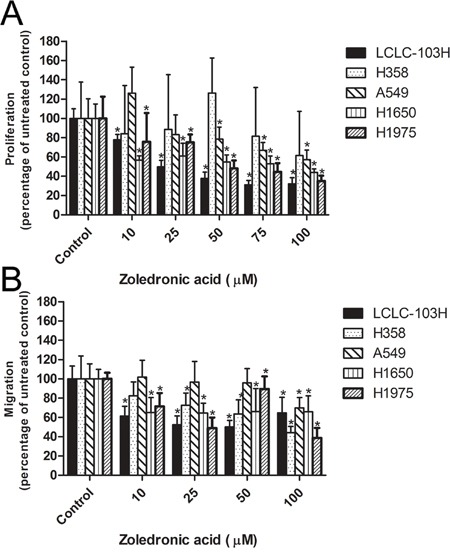
The effect of zoledronic acid on the proliferation and migration of human NSCLC cell lines Mutant KRAS-expressing H358 and A549 showed resistance against zoledronic acid, while the treatment reduced the proliferation capacity of cell lines with wild type KRAS **A.** The inhibitory effect of zoledronic acid was less dependent of KRAS-status **B.** (Data are mean ± SD, n=6). *: p<0.05 to untreated control.

As detailed above, H1650, H1975 and LCLC-103H cell lines express wild type KRAS, while A549 and H358 cells express mutant KRAS. After 48 hour treatment with different concentrations of zoledronic acid, A549 and H358 cells expressing mutant KRAS exhibited resistance. On the other hand, human NSCLC cell lines expressing wild type KRAS proved to be sensitive against zoledronic acid in dose dependent manner. The most sensitive cell line was LCLC-103H (wild type KRAS and EGFR). The maximal effect on the proliferation was approximately 40% of untreated control (100%) at 100 μM of zoledronic acid (Figure 2A).

### Zoledronic acid had low apoptotic potential in human NSCLC cell lines

After 48 hour treatment with different concentrations of zoledronic acid the apoptotic sub-G1 fractions were stained by propidium-iodide. Flow cytometric analysis showed that the LCLC-103H cell line was the most sensitive in this respect as well. Compared to the total events it was found that 100 μM of zoledronic acid increased the percentage apoptosis to almost 10%. In case of H1650 a relatively high level of basic apoptosis was measured (untreated control), and a significantly increased apoptotic level for zoledronic acid was not observable. In case of H1975 zoledronic acid induced statistically significant apoptosis. Since apoptosis remained in a very low range (1.61 ± 0.36% to 4.02 ± 0.03%) the biological role of this elevation is questionable. In case of the mutant KRAS-expressing H358 and A549 the level of apoptotic fraction showed no significant changes, in the latter cell line relatively high level of basic apoptosis was measured (Table 2).

**Table 2:** The *in vitro* effect of zoledronic acid on the apoptosis of human NSCLC-lines

	LCLC-103H	H1650	H1975	A549	H358
**Control**	1.06±0.35	6.33±0.42	1.61±0.36	6.04±1.65	2.42±1.24
**Zoledronic acid (25 μM)**	4.68±0.23*	8.72±0.4	1.73±0.28	8.43±2.55	2.82±0.55
**Zoledronic acid (50 μM)**	4.02±0.28*	7.19±0.17	2.94±0.06	10.6±2.51	4.77±1.46
**Zoledronic acid (100 μM)**	9.89±1.15*	5.75±1.47	4.02±0.03*	10.01±6.99	6.11±3.29

Statistically and biologically significant apoptosis was measured only in wild type EGFR- and wild type KRAS-expressing LCLC-103H. (±SD, n=3).

* p<0.05 to untreated control.

### Effect of the combination of zoledronic acid with cisplatin on the proliferation of human NSCLC cell lines

Cisplatin is a commonly used agent in the cytostatic therapy of NSCLC. Analyzing the *in vitro* proliferation assay H358 and H1650 showed relative resistance, H1975 and LCLC-103H proved to be sensitive, while A549 cell line was at the intermediate level. Except for A549, combining cisplatin with zoledronic acid the 50% inhibitory concentration values (IC50) were reduced in all of the studied human NSCLC cell lines, which meant additive effect (Table 3).

**Table 3:** The 50% inhibitory concentration (IC50) of cisplatin (in μM) on the proliferation of human NSCLC-lines in combination with zoledronic acid

	LCLC-103H	H1650	H1975	A549	H358
**Control**	0.19±0.01	4.39±0.21	0.35±0.15	1.27±0.62	8.08±1.51
**Zoledronic acid (10 μM)**	0.13±0.11*	1.62±0.4*	0.37±0.12	1.7±0.7	4.9±0.71*
**Zoledronic acid (25 μM)**	0.007±0.003*	0.29±0.11*	0.15±0.07*	1.75±0.89	2.59±0.32*
**Zoledronic acid (50 μM)**	<0.001*	0.26±0.12*	0.024±0.01*	0.89±0.43	1.27±0.26*

In combination, zoledronic acid reduced the concentration of cisplatin for the same inhibitory level (n=5).

* p<0.05 to untreated control.

### Effect of zoledronic acid on the *in vitro* migration of human NSCLC cell lines

Zoledronic acid can potentially target different regulatory small G proteins involved not only in the signalization of proliferation, but also in cell migration. In modified Boyden-chamber zoledronic acid inhibited migration of the human NSCLC cell lines, irrespectively of KRAS-status. However, in the case of KRAS-mutant cells, only higher concentrations proved to be effective. Detected effects were between 40 and 70% of untreated controls for all every human NSCLC cell lines (Figure 2B).

### The *in vivo* effect of zoledronic acid in subcutaneous xenograft model

**Figure 3:**
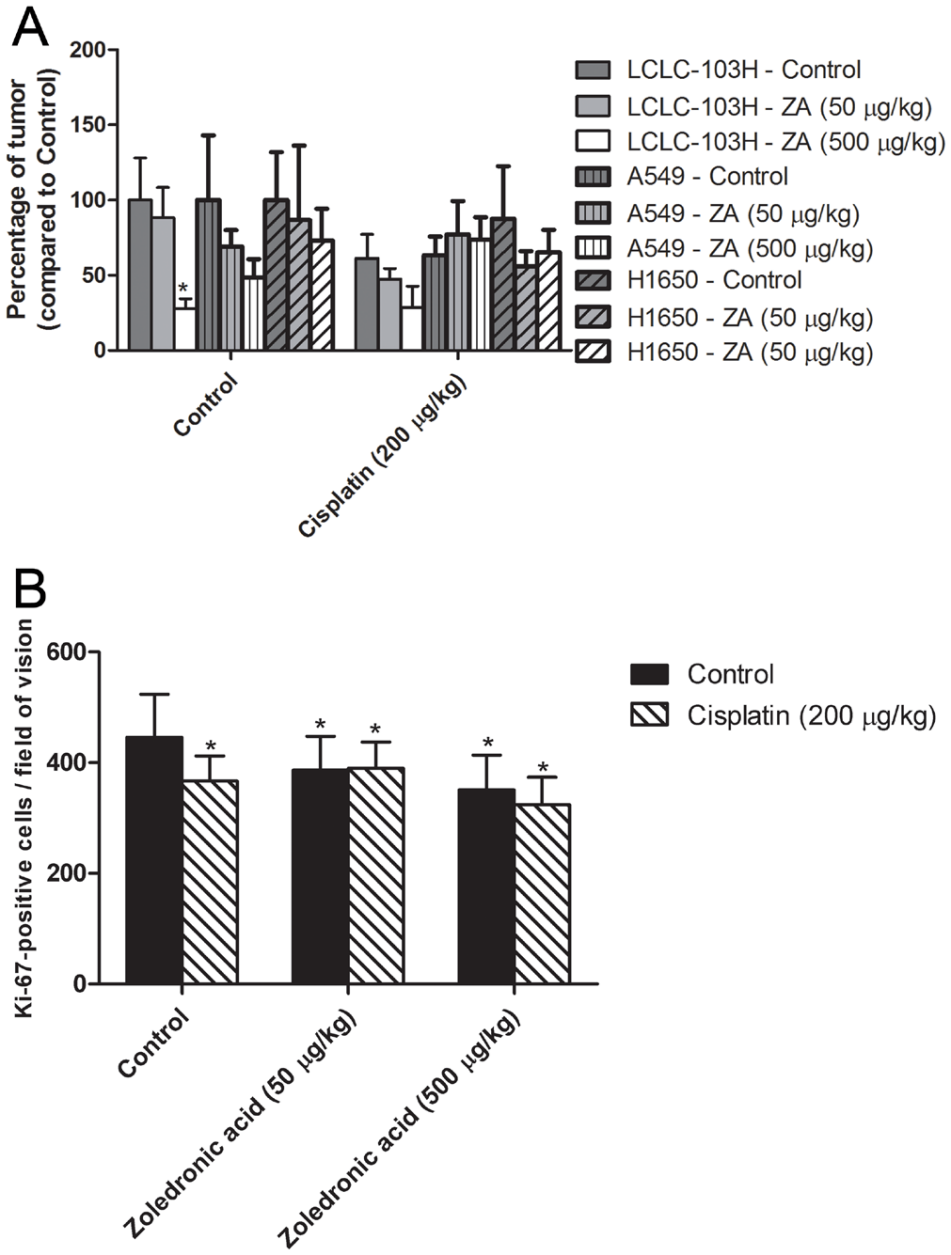
The effect of zoledronic acid on subcutaneous xenografts The highest dose of zoledronic acid (ZA) significantly inhibited the growth of subcutaneous LCLC-103H xenograft. However, zoledronic acid proved unable to increase the inhibitory effect of cisplatin significantly, albeit the tendency was clear. H358 xenograft tumor expressing mutant KRAS and H160 xenograft tumor expressing activating EGFR-mutation did not show significant effect to zoledronic acid or cisplatin **A.** (Data are means ± SEM, n=8; *: p=0.032 to solvent treated control.) Cisplatin significantly inhibited numbers of Ki-67-positive LCLC-103H cells in *in vivo* xenograft tumor, however zoledronic acid was incapable of boosting this effect in the applied doses **B.** (Data are means ± SEM, n=8*5; *: p<0.05 to solvent treated control.)

LCLC-103H, A549 and H1650 tumors were subcutaneously transplanted into SCID mice. Then after 21 days the xenografted mice were treated intraperitoneally weekly for three weeks with the human dose and a dose ten-fold the human dose of zoledronic acid (50 μg/kg and 500 μg/kg) combined with one-tenth of the human dose of cisplatin (0.2 mg/kg). Our results showed that the highest dose of zoledronic acid significantly reduced the weight of LCLC-L03H xenografts compared to that of the control group (P=0.032; Kruskal-Wallis test). In combination with cisplatin, zoledronic acid showed an evident tendency, which however did not prove to be statistically significant (Figure 3A). In concodance to our *in vitro* results, H358 xenograft tumor expressing mutant KRAS showed significant effect neither to zoledronic acid nor cisplatin. Surprisingly, zoledronic acid and cisplatin did not decrease the weight of H1650 xenograft tumor expressing activating EGFR-mutation.

To reveal the mechanism of the *in vivo* effect of zoledronic acid, xenograft tumors were analyzed by immunohistochemistry. The proportion of proliferating tumor cells was determined by Ki-67 labeling, which is a specific cellular marker for proliferation. In monotherapy cisplatin and zoledronic acid significantly inhibited *in vivo* the proportion of cycling LCLC-103H tumor cells, however in case of combinatory treatment such an effect cannot be detected (Figure 3B).

**Figure 4:**
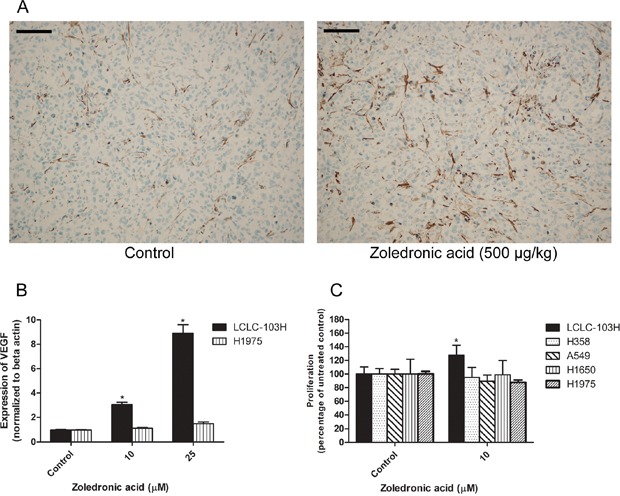
The effect of zoledronic acid on the *in vivo* vascularization of LCLC-103H xenograft and the *in vitro* modelling of this phenomenon Zoledronic acid enhanced the vascularization of LCLC-103H xenograft. Vessels were labeled by immunohistochemistry using anti-smooth muscle actin (SMA). Bars indicate 100 μM **A.** Zoledronic acid increased the *in vitro* expression of VEGF in LCLC-103H cell line shown by real time PCR, however did not affect the expression in H1975 cell line significantly (mean ± SD, n=3). Beta actin served as reference. *: p<0.05 to untreated control **B.** The proliferation of VEGF-sensitive KS-IMM (human Kaposi-sarcoma) cells was only induced by the supernatant of zoledronic acid-treated LCLC-103H cells. A549, H358, H1650 and H1975 did not show similar significant effect (mean ± SD, n=5). Zoledronic acid was applied on the non-small cell lung cancer lines for 48 hours, thereafter filtrated supernatants were applied on KS-IMM cells for another 48 hours **C.**

The *in vivo* effect of zoledronic acid on the vascularisation of human LCLC-103H xenograft was analyzed by vessel-specific SMA labeling (Figure 4A). SMA (smooth muscle actin) strains smooth muscle, which is a critical component in the wall of the vessels. Compared to solvent treated control, zoledronic acid significantly induced vascularization of subcutaneous xenograft at 500 μg/kg dose (39.87 ± 12.84 vs. 51.67 + 16.98 positive vessels per field of vision, respectively; data are mean ± SD, P=0.015, Mann-Whitney's u test).

### Mechanism of the vascular effects of zoledronic acid in *in vitro* models

VEGF is one of the main regulators of angiogenesis. The *in vitro* expression of VEGF in H1975 and LCLC-103H human NSCLC was measured by real-time PCR. In serum-free conditions, 48 hour administration of zoledronic acid in 10 or 25 μM significantly increased the expression of VEGF in wild type KRAS-expressing LCLC-103H cells, while in mutant KRAS carrier H1975 the effect was not significant, albeit a tendency was observable (Figure 4B).

In previous studies it was shown that VEGF induced the proliferation of human Kaposi sarcoma cells [29, 30]. Since we detected increased level of VEGF in the case of zoledronic acid-treated LCLC-103H cells, we have generated 10 μM zoledronic acid-induced supernatants in serum-free conditions and administered to KS-IMM cells for 48 hours. Compared to untreated control cells, we have detected that zoledronic acid-induced supernatant increased proliferation of human KS-IMM cells in case of supernatant of the zoledronic acid-treated LCLC-103H cells (127.45 + 17.75 % of the control; mean ± SD, P=0.0002). Meanwhile, zoledronic acid did not affect the proliferation of KS-IMM cells directly (data not shown). These findings strengthened the theory of the *in vivo* vascularization-inducing effect of zoledronic acid. On the other hand, A549, H358, H1650 and H1975 did not show similar effect significantly (Figure 4C).

## DISCUSSION

For a long while, the mutation of KRAS-oncogene has been in the focus of scientific and clinical interest. The most frequent mutation of KRAS is localized in codon 12 (G12C), which affects approximately one third of the experimentally used NSCLC-lines and 30% of patients with lung cancer [6, 31]. The active wild type K-Ras protein is localized in the plasma membrane, which requires prenylation by farnesyltransferases or geranylgeranyltransferases, [9]. Bone metastasis inhibitor zoledronic acid has the potential to prevent prenylation of small-G proteins by inhibition of farnesyl-diphosphate synthase [12], suggesting the anticancer effect of the agent not only on the metastases, but directly on the primary tumor as well. Numerous preclinical works provided evidence on the anti-proliferative effect of zoledronic acid in breast cancer, pancreatic cancer, prostate cancer, small and non-small lung cancers under both *in vitro* and *in vivo* conditions [16–20, 23–25], however, the exact explanation of the efficacy is not yet been revealed.

Our systematic analysis demonstrated that the critical target of zoledronic acid was K-Ras protein: in human NSCL cell lines prenylation of wild type Ras (H1975, LCLC-103H) is inhibited by zoledronic acid, leading to reduced proliferation capacity, thus treatment in combination resulted higher susceptibility against the standard cytotoxic drug, cisplatin. On the other hand, exon2/codon12-mutant Ras (in H358 and A549) does not need prenylation for its activity, therefore usage of zoledronic acid was ineffective from this aspect. Surprisingly, zoledronic acid did not inhibit prenylation of K-Ras protein in H1650 cells expressing activating EGFR-mutation, which suggested that this cells did not prefer K-Ras pathway in the signalization of EGFR.

Previous preclinical studies described zoledronic acid as an apoptotic agent in human breast cancer and pancreatic cancer cell lines [16, 17]. However, in our *in vitro* model zoledronic acid did not induce apoptosis in human NSCLC lines, similarly to other works focusing on NSCLC [25, 32]. In our xenograft tumors immunohistochemistry proved reduced number of Ki-67-positive cells after treatment with zoledronic acid, which corroborates the *in vitro* findings of Chang et al. and Li et al. that the inhibitory effect of the drug emerges rather through the blockade of cell cycle than apoptosis [25, 32].

Numerous preclinical data are available on the synergistic effect of zoledronic acid with cytostatic agents, such as paclitaxel, antracyclins, cisplatin, gemcitabine, and topoisomerase inhibitors in human cancer cell lines including SCLC [20, 22–24, 33]. Previously in NSCLC only one *in vitro* study proved that zoledronic acid enhanced the cytotoxicity of cisplatin in the A549 cell line [34], and other works provided data for the synergistic effect of zoledronic acid with EGFR-based therapy [25]. However, this work is the first to demonstrate the *in vivo* enhancing effect of zoledronic acid with the most widely used cytotoxic agent cisplatin. Similarly to our work, a previous study showed synergistic effect in A549 cells expressing G12C-mutant KRAS, however that set of experiments focused only on proliferation and apoptosis [34]. According to that paper 100 μM zoledronic acid showed 50% cytotoxicity on its own, which contradicts our data. Since neither we and nor other previous works have observed any significant apoptosis inducing capacity of zoledronic acid in human NSCLC cell lines, we assume a special experimental model in the background.

Another interesting aspect of the *in vivo* effect of zoledronic acid is angiogenesis. Previous preclinical and clinical studies proved that zoledronic acid could be an anti-angiogenic agent, which could inhibit proliferation of HUVEC endothelial cells, suppress expression of VEGF in A549 NSCLC line (with G12S-mutant KRAS), and cause decreased level of VEGF in the circulation of cancer patients [19, 35]. On the contrary, our work demonstrated elevated VEGF-expression *in vitro* in NSCLC cells carrying wild type KRAS, and improved vascularization of xenograft tumors *in vivo* treated by zoledronic acid. However, based on our *in vitro* results, increased proliferation of vascular cells were only observed in the case of double wild type cells, cells with different genetic background did not show this effect. Although numerous paper stated zoladronic acid as an anti-angiogenic agent, in parallel with our work there were some that proved that in certain environmental conditions zoledronic acid could induce VEGF expression. Zafar et al. confirmed that zoledronic acid induce VEGF expression in human gingival fibroblast [36]. Furthermore, other inhibitory agents of prenylation, statins induced expression of VEGF [37]. Those papers suggested that in the background of induction of VEGF is not related to the mevalonate pathway. Since the induction could be attenuated by PI3K (phosphoinositid 3-kinase) inhibitor LY294002, other signalization mechanism (e.g. PI3K-pathway) was suggested.

Previous studies showed the potential benefit of angiogenesis in subcutaneous *in vivo* models on HT25 colorectal and A431 squamous cell cancer xenograft, since increased vascular density promoted the penetration of cytostatic agent [38]. In concordance, our *in vivo* model with LCLC-103H the vascularization effect of zoledronic acid could contribute to the cytostatic effect of cisplatin. It is a well known fact that intratumoral blood vessels are frequently immature and leaky causing intratumoral hypoxia and resistance to cytostatic effects of drugs. Several clinically used “angiogenesis inhibitors” operate through vessel normalization, by improving better drug penetration into tumors [39]. Here we have used a pericyte marker for blood vessel identification. The observed paradoxical effect of zoledronic acid on VEGF in wild type KRAS expessing NSCLC tumor and its consequences on improved vascularization/vessel stabilization may easily follow similar biological trends.

According to other studies, nitrogen-containing bisphosphonates inhibited adhesion of breast cancer and prostatic cancer cells to the elements of the extracellular matrix, which prevented invasion and metastasis formation of cancer cells [40]. Irrespectively of KRAS-status, Zoledronic acid reduced migratory capacity of all the applied NSCLC lines, which suggests the inhibition of other small G proteins involved in motility signal (such as Rho, Rac, Cdc42) by preventing posttranslational prenylation.

## MATERIALS AND METHODS

### NSCLC cell cultures

The genetic background of the applied human NSCLC lines is summarized in Table 1. A549 adenocarcinoma expressed exon2-mutant KRAS (G12S), H358 human bronchioalveolar carcinoma cell line expressed exon2-mutant KRAS (G12C), both with wild type EGFR. LCLC-103H human large cell line expressed wild-type KRAS and wild-type EGFR. H1650 and H1975 human adenocarcinoma cell lines expressed wild-type KRAS, H1650 had an activating exon19 EGFR-mutation, while H1975 showed resistance against EGFR-TKIs due to exon21 mutations. All the cell lines were grown in medium RPMI-1640 (Sigma-Aldrich, St. Louis, MO) supplemented with 5% fetal calf serum (FCS) (Sigma-Aldrich) and 1% penicillin-streptomycin (Sigma-Aldrich).

### Western-blot analysis

After 48 hour incubation with 25 μM zoledronic acid or control medium, culture media were replaced with ice-cold phosphate buffered saline, and cells were removed from the flask surface using a cell scraper. The samples were then centrifuged for 5 minutes at 300 g on 4°C to precipitate the cells. After discarding the supernatant, buffer A was added [10 mM NaCl, 1.5 mM MgCl2, 10 mM Tris (pH 7.4) containing protease inhibitors (8 μg/mL aprotinine, 10 μg/mL leupeptine, 50 μg/mL PMSF and 2 mM DTT)]. The samples were disrupted with a Dounce homogenizer (80 strokes; Sigma-Aldrich). Following homogenization, Buffer B [525 mM mannitol, 175 mM sucrose, 12.5 mM Tris (pH 7.4), and 2.5 mM EDTA containing the same concentration of protease inhibitors as buffer A] was added in a ratio of 4:10 homogenate/buffer B. To obtain membrane and cytosol fractions, total lysates were centrifuged at 100,000 g at 4°C for 60 min in a 70.1 Ti rotor (Beckman), Indianapolis, IN). The organelle and membrane samples were diluted 1:1 by 2x Laemmli buffer (Sigma-Aldrich), and stored at −80°C until further use. The whole separation procedure was performed on ice to avoid protein degradation.

Protein content of the samples was measured according to the method of Lowry [41]. The proteins were separated in a 7.5% SDS-polyacrylamide gel, then transferred to a PVDF membrane (Bio-Rad, Hercules, CA), using a wet electroblotting apparatus according to the manufacturer's protocols. Primary antibodies used during western blot: K-Ras (ab55391, Abcam, Cambridge, MA) Na/K-ATPase (BML-SA247-0100, Enzo Life Sciences, Farmingdale, NY). Horseradish-peroxidase conjugated anti-mouse IgG secondary antibody was purchased from Jackson Immuno Research Laboratories. Immunoblots were revealed by enhanced WesternBright chemiluminescence system (Advansta, Menlo Park, CA). Expression of K-Ras was compared to the level of Na/K-ATPase.

### Cell proliferation assay

Cell suspension containing 5×10^3^ viable cells/well was plated onto 96-well dishes and allowed to attach at 37°C in 5% CO_2_ atmosphere in RPMI-1640 medium (Sigma-Aldrich) supplemented with 5% FCS. In case of the LCLC-103H cell line, 2.5×10^3^ viable cells/well was applied. After 24 hours, cells were exposed to different concentrations of cisplatin (“Ebewe” cisplatin, Ebewe Pharma, Austria) and zoledronic acid (Zometa®, Novartis Pharmaceuticals Inc., Switzerland) for 48 hours. The effects of the treatments were analyzed by MTT (Sigma-Aldrich) colorimetric assay [42].

The results of three independent experiments were transferred to 50% inhibitory concentrations (IC50), which were calculated by Dose-Effect Analysis with Microcomputers software (Elsevier-Biosoft, Cambridge, UK).

### Flow cytometric measurement for apoptosis

Cell suspension containing 3×10^5^ viable cells/well was plated in 6-well dishes and allowed to attach for 24 hours at 37°C in 5% CO_2_ atmosphere in RPMI-1640 medium (Sigma-Aldrich) supplemented with 5% FCS (Sigma-Aldrich). In case of the LCLC-103H cell line we applied 1.5×10^5^viable cells/ml. After the medium was changed (5% FCS) the cells were exposed to different concentrations of zoledronic acid for 48 hours. Then the treated cells were detached with 0.02% EDTA, washed with PBS and then fixed with 70% ethanol. According to the protocol of the manufacturer, after several hour incubation period with propidium-iodide and RNAse (CyStain PI Absolute T, Partec, Germany), we quantified the DNA in the cells by flow cytometer (CyFlow, Partec). The percentage of apoptotic cells was shown by the sub G1 fraction which was analyzed using FlowMax software (Partec) [43].

### Modified Boyden-chamber migration assay

Cell migration procedure was assayed by a method reported previously by Albini et al [44]. We used 96-well CXF8 plate (polycarbonate filter with 8 μm pore size, Neuroprobe Inc., Cabin John, MD). Previously cultured cells were harvested with 0.02% EDTA, washed twice with serum free medium and resuspended at 10^6^cells/ml in medium with 0.1% BSA containing treatment material. In case of the LCLC-103H cell line we started with 0,5×10^6^ viable cells/ml. The cell suspension (20 μl) was placed onto the membrane (Neuroprobe) in the presence of the treatment agent and the lower compartment was filled with 30 μl of fibronectin (100 μg/ml) (Sigma-Aldrich). Cells were allowed to migrate for 24 hours at 37°C in a humidified atmosphere containing 5% CO_2_, then cells on the upper surface of the filter were removed mechanically, the membranes were stained with toluidin blue, and the cells in fields of vision were counted manually under light microscope.

### Subcutaneous xenograft model

Previously *in vivo* subcutaneously cultured LCLC-103H, A549 and H1650 human NSCLC tumors were subcutaneously transplanted in male BALB/c SCID mice; the animals were used at 2-3 months of age. Sizes of 16 mm^3^ tumor per animal were implanted subcutaneously during anaesthetized state. At day 21, after randomization animals were treated intraperitoneally with the human dose and a dose ten-fold the human dose of zoledronic acid (50 μg/kg and 500 μg/kg) combined with one-tenth of the human dose of cisplatin (0.2 mg/kg) weekly for three weeks. Controls received 0.9% NaCl in the same volume. At day 41 animals were sacrificed by Nembutal (Serva, Heidelberg, Germany) overdose. Subcutaneous tumors were removed and the tumor weights were measured. The xenograft tumors were fixed in 10% neutral buffered formalin.

### Immunohistochemistry

Monoclonal mouse antibody against human Ki-67 (DAKO, Denmark) and human smooth muscle actin (SMA) (DAKO) were applied. The routinely formalin-fixed xenograft tumors were dehydrated in a graded series of ethanol, infiltrated with xylene and embedded into paraffin at a temperature not exceeding 60°C. Three to four micron thick sections were mounted on Superfrost slides (Thermo Shandon, Runcorn, UK) and were manually deparaffinized. To block endogenous peroxidase activity, slides were treated for 5 min at room temperature with 3% H_2_O_2_ in methanol. Slides were immersed in 0.05 mM citrate buffer (pH=6) and exposed to 750 W microwave for 3×5 min (MFX-800-3 automatic microwave, Meditest, Budapest, Hungary).

Slides were primarily treated with antibody against human Ki-67 (1:40) or SMA (1:100) and incubated for 1 hour at room temperature. After washing with phosphate-buffer-saline, secondary antibody Biotinylated Link (Dako) was applied and incubation occurred for 10 minutes at room temperature. After washing periods for visualization, a standard avidin-biotin peroxidase technique (ABC system, DAKO) was used with diaminobenzidine as chromogen.

The Ki-67-positive tumor cells or SMA-positive vessels per fields of vision were counted manually under light microscope (200-fold magnification), 5 fields of vision per tumor were evaluated.

### Effect of zoledronic acid-treatment on the VEGF-production of human lung cancer cell lines

Previously cultured human lung cancer H1975 and LCLC-103H cells were plated on 25 cm^2^ tissue culture flasks (5×10^5^cells/flask) in RPMI supplemented with 5% FCS and were allowed to grow. After two days cells were treated for 48 hours with 10 or 25 μM of zoledronic acid in serum-free conditions. The treated cells were compared to untreated control.

Total RNA was extracted from the cells using TRI Reagent™ (Sigma-Aldrich) according to the manufacturer's protocol. Possible DNA contamination was eliminated using TURBO DNA-free™ kit (Ambion^®^). Three μg of total RNA were reverse transcribed from each sample using deoxy-NTPs (Promega, 0.5 mM each), a mixture of random primer and oligo dT (Promega, final concentration 3 μM), RNasin^®^ ribonuclease inhibitor (Promega, 20 U/reaction), M-MLV RT 5x Reaction Buffer (Promega, containing 250 mM Tris-HCl, pH 8.3, 375 mM KCl and 15 mM MgCl_2_ and 50 mM DTT) and M-MLV Reverse Transcriptase RNase H Minus, Point Mutant (Promega, 200 U/reaction). RNA-solutions with the oligos were pre-incubated for 5 min at 70°C, then after cooling down and adding the dNTPs and enzymes the incubation continued for 10 min at 25°C, then 50 min at 45°C. To inactivate the reaction the mixture was heated to 70°C for 15 min.

The real-time PCR analysis was standardized by co-amplifying the genes of interest with the housekeeping gene β-actin (Applied Biosystems Taqman probe: Hs03023880_g1). The real-time PCR reaction was run on the Applied Biosystem's 7500 Real-Time PCR System. The reaction mixture contained the followings: VEGF Taqman probe (Hs00900054_m1, Applied Biosystems), TaqMan® Universal PCR Master Mix (Applied Biosystems) and 2 μg cDNA. No template control (containing water) was used as negative control for the different primer-pairs. The cycling parameters were 50°C (2 min), 95°C (10 min), 40 cycles of 95°C (15 sec) and 60°C (1 min). The starting quantity of gene expression in the samples was determined by comparison of unknowns to a standard curve generated from a dilution series of template DNA of known concentrations, and normalized to their own β-actin expression.

### Effect of the supernatant of treated LCLC-103H cells on the proliferation of KS cells

KS-IMM (human Kaposi sarcoma cell line) [45], LCLC-103H, H358, A549, H1650 and H1975 cells were previously cultured in RPMI-1640 medium (Sigma-Aldrich) supplemented with 5% FCS (37°C and 5% CO_2_ atmosphere). Cell suspension containing 5×10^4^/well viable LCLC-103H, H358, A549, H1650 and H1975 cells were plated in 6-well dishes and allowed to attach. After 24 hours the cells were exposed to 10 μM of zoledronic acid for 48 hours. The filtrated supernatant of treated and untreated cells was placed onto cultured KS-IMM cells in 96-well dishes. Previously the KS-IMM cells were allowed to attach for 48 hours in the concentration of 5×10^4^viable cells/ml in 96-well dishes. We treated the KS-IMM cells with supernatant for 48 hours in serum-free conditions. The effects were analyzed by MTT (Sigma-Aldrich) colorimetric assay, as detailed above.

### Statistics

To determine statistical differences between groups, ANOVA was used with *post hoc* Scheffé-test, where parametric methods were available. In case of the animal experiments we used non-parametric Kruskal-Wallis test. Statistical significance was determined when Ps were <0.05. Statistical analysis was performed by Statistica 12.0 (StatSoft, USA, Tulsa, OK).

## SUPPLEMENTARY FIGURE



## Abbreviations

DNA: deoxyribonucleic acid
EGFR: epidermal growth factor receptor
FCS: fetal calf serum
FPP: farnesyl diphosphonate
GTP: guanosine triphosphate
Na/K-ATPase: sodium-potassium adenosine triphosphatase
NBP: nitrogen-containing bisphosphonate
NSCLC: non-small cell lung cancer
PCR: polymerase chain reaction
PI3K: phosphoinositide 3-kinase
PLC: Phospholipase C
Ras: protein product of RAS gene
RAS: rat sarcoma gene
RNA: ribonucleic acid
SCLC: small cell lung cancer
SD: standard deviation
SEM: Standard error of mean
SMA: smooth muscle actin
TKI: tyrosine kinase inhibitor
VEGF: vascular epithelial growth factor
ZA: zoledronic acid.
