# Supplementary material for: KRAS-mutation status dependent effect of zoledronic acid in human non-small cell cancer preclinical models
Source: Oncotarget. 2016 Oct 21;7(48):79503–14. doi: 10.18632/oncotarget.12806 (PMC5346731; doi:10.18632/oncotarget.12806)
Supplement: Supplementary file 1 [file oncotarget-07-79503-s001.pdf]

KRAS-mutation status dependent effect of zoledronic acid in human non-small cell cancer preclinical models

SUPPLEMENTARY FIGURE

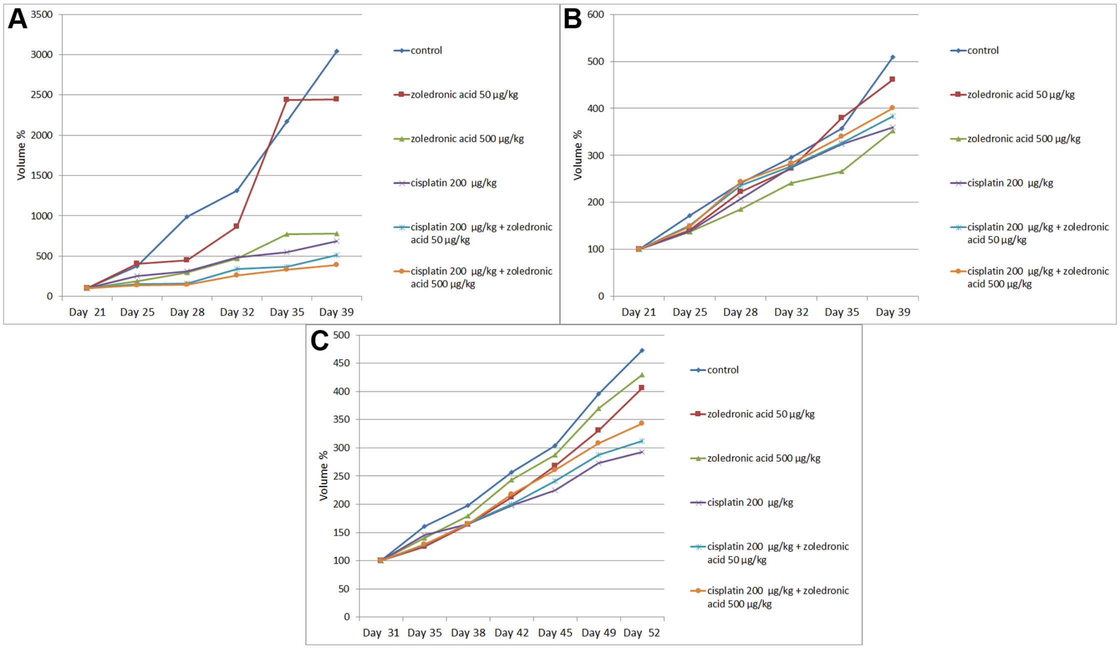

Supplementary Figure S1: Relative change of human subcutaneous xenograft tumors during treatment with zoledronic acid and cisplatin. Compared to the initial day of treatment, time course of tumor growth revealed that zoledronic acid was effective only on LCLC-103H xenograft, which effect could not be enhanced by cisplatin **A.** H358 xenograft tumor expressing mutant KRAS **B.** and H1650 xenograft tumor expressing activating EGFR-mutation **C.** did not show significant effect to zoledronic acid or cisplatin. (Data are mean of percentage compared to the initial volume of tumor on the day of first treatment, n=8).
